# Medical causes of admissions to hospital among adults in Africa: a systematic review

**DOI:** 10.3402/gha.v6i0.19090

**Published:** 2013-01-08

**Authors:** Anthony O. Etyang, John Anthony Gerard Scott

**Affiliations:** 1 Department of Epidemiology and Demography, Kenya Medical Research Institute/Wellcome Trust Research Programme, Kilifi, Kenya; 2 Nuffield Department of Clinical Medicine, University of Oxford, Oxford, England

**Keywords:** adults, Africa, medical admissions, health, transition, ICD, cause of death

## Abstract

**Background:**

Despite the publication of several studies on the subject, there is significant uncertainty regarding the burden of disease among adults in sub-Saharan Africa (sSA).

**Objectives:**

To describe the breadth of available data regarding causes of admission to hospital, to systematically analyze the methodological quality of these studies, and to provide recommendations for future research.

**Design:**

We performed a systematic online and hand-based search for articles describing patterns of medical illnesses in patients admitted to hospitals in sSA between 1950 and 2010. Diseases were grouped into bodily systems using International Classification of Disease (ICD) guidelines. We compared the proportions of admissions and deaths by diagnostic category using χ^2^.

**Results:**

Thirty articles, describing 86,307 admissions and 9,695 deaths, met the inclusion criteria. The leading causes of admission were infectious and parasitic diseases (19.8%, 95% confidence interval [CI] 19.6–20.1), respiratory (16.2%, 95% CI 16.0–16.5) and circulatory (11.3%, 95% CI 11.1–11.5) illnesses. The leading causes of death were infectious and parasitic (17.1%, 95% CI 16.4–17.9), circulatory (16%, 95% CI 15.3–16.8) and digestive (16.2%, 95% CI 15.4–16.9). Circulatory diseases increased from 3.9% of all admissions in 1950–59 to 19.9% in 2000–2010 (RR 5.1, 95% CI 4.5–5.8, test for trend p<0.00005). The most prevalent methodological deficiencies, present in two-thirds of studies, were failures to use standardized case definitions and ICD guidelines for classifying illnesses.

**Conclusions:**

Cardiovascular and infectious diseases are currently the leading causes of admissions and in-hospital deaths in sSA. Methodological deficiencies have limited the usefulness of previous studies in defining national patterns of disease in adults. As African countries pass through demographic and health transition, they need to significantly invest in clinical research capacity to provide an accurate description of the disease burden among adults for public health policy.

The health of adults in sub-Saharan Africa (sSA) is becoming an increasingly important priority in global health policy (1, 2). Recent improvements in the survival of children (3–5) coupled with a reduction in total fertility mean that the proportion of the population that is adult is increasing rapidly, beginning with young adults (6). In any society adults are the economically productive age group. The survival of children is closely linked to that of adults and to maintain the gains made in reducing child mortality, the causes of early adult mortality need to be identified (7). Recent studies show that levels of adult mortality (i.e. death between the ages of 15 and 60) are 4–40 times higher in sSA than in developed countries (3, 8–11).

The pattern of illnesses responsible for the high mortality among adults in sSA has not been well characterized (2, 9–13). The World Health Organization (WHO) predicts that by 2020, the causes of disease and death in sSA will have undergone a significant shift towards endemic non-communicable diseases and away from infectious diseases (14). This shift will necessitate changes in the deployment of resources, both human and physical, to deal with new health challenges (15, 16). Currently very few health systems in low- and middle-income countries rely on research evidence for guiding policy interventions (17). As this transition occurs, accurate data on the burden of illnesses will be needed as there will be a double burden of endemic non-communicable and residual communicable diseases (2, 15, 18).

Community-based studies provide an accurate picture of the profile of the diseases of adults because they minimize the bias, inherent in hospital-based studies, of variable access to health care (19, 20). However, these surveys are expensive undertakings, and useful information could be gained from analysis of well-conducted routine population and clinical surveillance activities. There are currently substantial efforts to improve vital event registration including the introduction of verbal autopsy methods to better describe causes of death. However, the shortcomings of health facility-based studies have not been systematically analyzed in order to improve future work. Here we report a systematic review of descriptive studies of medical illnesses in adults admitted to hospitals in sSA between 1950 and 2010 to determine the causes of admission and death and to characterize the methodological features of such studies that are likely to yield useful data in the future.

## Methods

### Literature search

We searched PubMed, African Index Medicus, *African Journals OnLine* (AJOL) and EMBASE for articles describing the pattern of disease among adults admitted to hospital in Africa. The Internet search was performed on 3 July 2011 using the search filter ‘(medical OR hospital) AND admissions AND adults AND Africa’. We screened titles and abstracts of articles that met specific inclusion criteria and read those that were available online or from academic libraries that we had access to. We reviewed reference lists of selected articles to identify additional secondary articles not found during the online search. Our review was limited to articles either written in or translated into English.

Studies were eligible for inclusion in the review if they were conducted among adult patients admitted to hospitals in sSA between 1950 and 2010. We excluded studies if data on adult admissions could not be extracted from them, if the study was conducted during a period of war, if the articles focused on specific age sub-groups (e.g. adolescents, geriatrics), or if the focus of the study was on a single factor as a cause of admissions to hospital, for example, HIV, injuries or obstetric admissions.

### Data extraction

We designed a data extraction form and entered the data from each paper into the form, which was later cross-checked for accuracy. The following data were collected from each study: country where the study was performed, study period, type of hospital, and number and causes of admissions and deaths. The diseases reported were grouped into body systems according to International Classification of Diseases (ICD) guidelines (21). We used the χ^2^ test to compare proportions of observations in each diagnostic category. We calculated risk ratios for disease changes over time by dividing the proportion of admissions and deaths in each category per decade by the corresponding value in the baseline period (1950–9) and we tested the trend in the sub-category with greatest changes by using the χ^2^ test for linear trend. p Values reported are two-sided and proportions reported with 95% confidence intervals (CIs). Data were analyzed using STATA^®^ v11.

### Review of methodological quality

We evaluated each paper using a uniform set of questions designed to gauge the quality of the studies. The criteria were adapted by the authors from the principles of descriptive epidemiology (22, 23) and the STROBE statement (24). The criteria and their justification are outlined in Table 1. Each criterion was assigned relative weights based on the authors’ assessment of their influence on study quality. We calculated the sum of all criteria scores to provide an overall methodological score for each study. We performed a methodological sensitivity analysis for the cause of death analysis by comparing the causes derived from studies ranked above the 75th percentile in their methodological score with the causes in the baseline analysis.

**Table 1:** Weighted criteria used in assessing methodological quality of studies^a^

Criterion	Justification
*What was the study design?* *Weight: 4 points*	Case ascertainment and record keeping is likely to be superior in prospective studies.
*Were case definitions used to define diseases reported?* *Weight: 6 points*	Comparisons can only be made between different studies if they adhered to standard case definitions, preferably those derived from the World Health Organization. For example, failure to use standard definitions has led to over-estimation of the burden of malaria as many patients with febrile illness in Africa are incorrectly classified as having malaria (72).
*Were the diseases coded using the International Classification of Diseases (ICD)?* *Weight: 4 points*	Established in 1948, the ICD is the international standard diagnostic classification for all general epidemiological, many health management purposes and clinical use (73). Due to the paucity of diagnostic facilities available in most settings in sub-Saharan Africa, we checked for the broad diagnostic categories of illnesses within the ICD, limiting classification to three character subcategories. Malaria for example would be classified as B50.
*Did the hospital have a defined set of criteria for admitting patients?* *Weight: 6 points*	The pattern of illness in any study is a function of (a) the pattern of disease in the local community and (b) the probability of admission with a particular condition once acquired. Patterns and policies of admission can therefore bias hospital data, more or less, as an indicator of the community burden of disease. Some hospitals only accept referrals, while others may have special units with certain diseases such as tuberculosis.
*What was the cadre of staff leading care provision at the hospital?* *Weight: 4 points*	It is recognized that there is a significant shortage of medical staff in Africa (50, 74) and that the accuracy of diagnoses made are dependent on the level of training of the personnel available (75). We categorized hospital staff into 3 groups of increasing training and competency and weighted studies on this basis: 1 point: Non-physician health workers – composed of clinical officers and physician assistants who on average have had 3–4 years of medical training (76). 2 points: Medical Officers – staff who had a basic medical degree (5–6 years of training at university) with no specialization or who are in the process of specializing, variously referred to as Medical Officers, House Officers, or Residents. 4 points: Consultants – staff who had an advanced medical training beyond the basic medical degree and were providing specialist care. For each study, the highest level of staff available at the hospital during the study period was recorded.
*What diagnostic facilities were available?* *Weight 6 points*	The availability of diagnostic facilities is critical for diagnosing some illnesses and facilities lacking basic diagnostic equipment would not be able to accurately describe disease patterns apart from making syndromic diagnoses. Studies could fall into one of 3 categories, weighted as shown: 2 points: health facilities that had the most basic diagnostic facilities, limited to microscopy, urinalysis and no imaging capability. 4 points: Health facilities in this category had as a minimum access to basic imaging equipment such as X-ray, laboratory tests including full blood count and chemistry panels. 6 points: These facilities had access to more advanced imaging and laboratory tests relative to other hospitals in the region. Tertiary referral and teaching hospitals were expected to fall under this category.
*Were case fatality/mortality rates reported?* *Weight: 6 points*	Mortality rates give an indication of the severity of the illnesses seen although this is subject to confounding by factors such as availability of medications and quality of care given. In addition, if mortality data are given by diagnostic category this allows us to estimate the ranking of disease burden both at the level of hospital admission and total (in-patient) mortality. We examined articles for reporting on case fatality or overall in-hospital mortality.

a Criteria and weights were developed by the authors, see text.

## Results

**Fig. 1:**
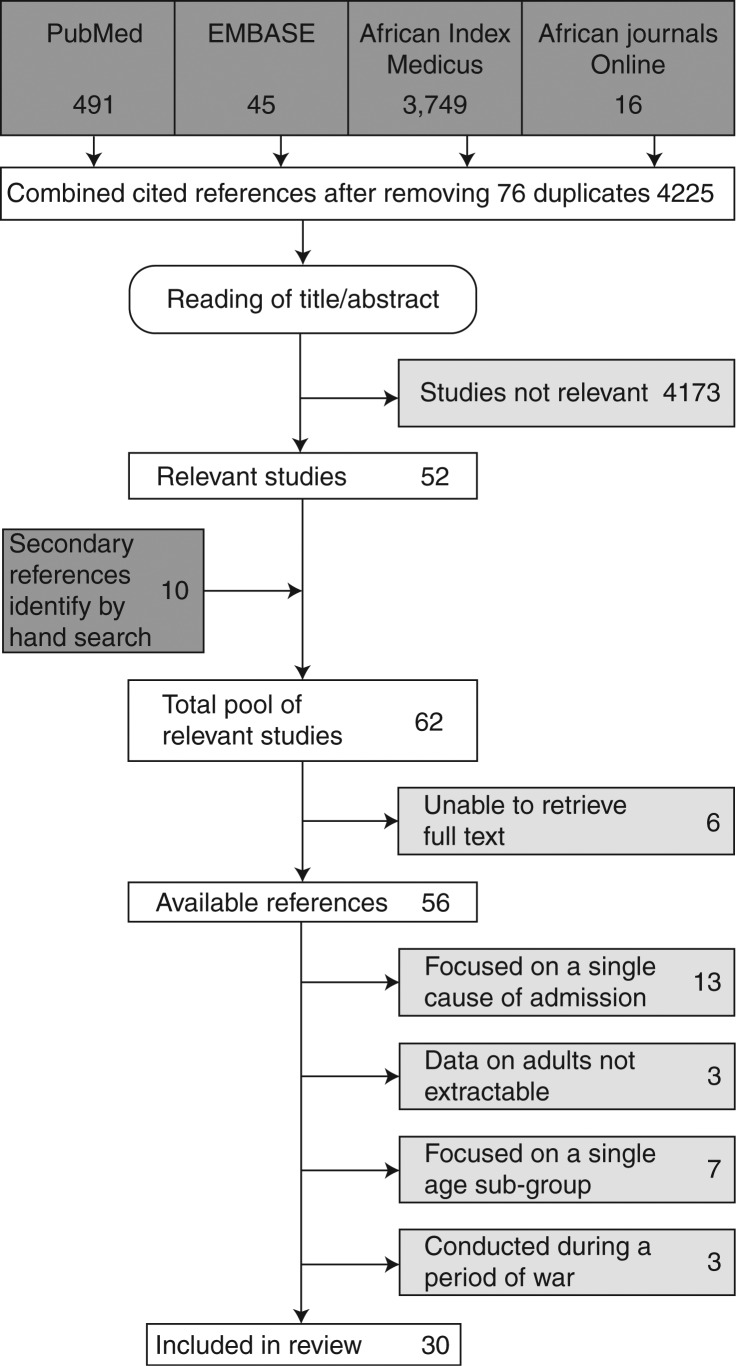
Search strategy and results.

**Fig. 2:**
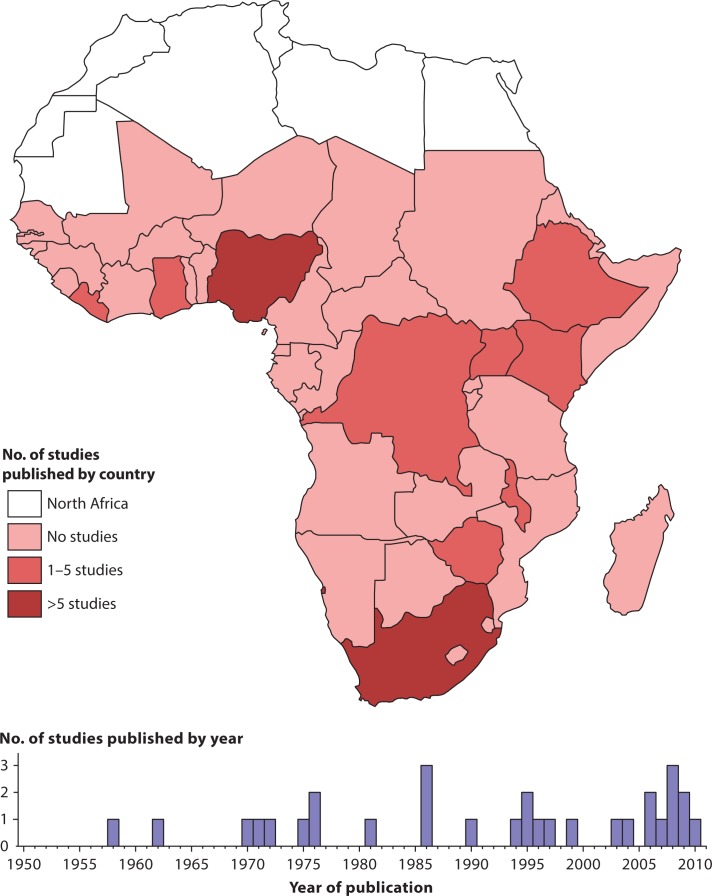
Map of sub-Saharan countries included in review and number of publications by year.

The online and hand-based search identified 56 relevant and obtainable full-text articles; 26 of these met the exclusion criteria leaving 30 full-text articles for inclusion in the review (Fig. 1). The geographic distribution of these studies by country and the number of studies published per year are shown in Fig. 2. The methodological characteristics of the studies included in the review are summarized in Table 2. The supplementary appendix (Table S1) lists the studies that were excluded, with reasons.

**Table 2:** Characteristics of studies included in review

Methodological quality^a^													

1st Author	Country	Year	No. of patients	Proportion female (%)	Case definitions provided? (6)	Study design (4)	ICD Coding? (4)	Cadre of staff (4)	Diagnostic facilities (6)	Admission criteria specified? (6)	Mortality rates reported? (6)	Score (x/36)	Methodology score%
Adekunle (36)	Nigeria	2004	104	50	−	R	+	C	III	−	+	23	64
Adetuyibi (42)	Nigeria	1960–73	4,568	45	−	R	−	C	III	+	+	25	69
Agomuoh (77)	Nigeria	2000–04	3,294	42	−	R	−	C	III	?	−	15	42
Amsel (26)	Ethiopia	1994–95	1,139	49	+	P	−	C	III	−	+	17	47
Bardget (78)	Zimbabwe	1992, 2000	2,674	52	−	P	−	C	III	?	+	28	78
Barr (27)	Kenya	1970–72	784	43	−	P	−	C	III	−	+	25	64
Brown (28)	Malawi	1972–73	2,289	43	−	P	−	C	III	−	+	23	64
Dean (37)	S. Africa	1984	1,571	40	−	R	−	C	III	−	+	23	56
Edginton (29)	S. Africa	1968–70	485	100	−	P	−	M	II	−	−	20	36
Gill (76)	S. Africa	1981, 1990	1,052	N/A	−	R	−	C	III	−	−	13	42
Griffiths (44)	S. Africa	1959, 1977	1,118	46	−	R	−	M	II	−	−	15	28
Harries (80)	Malawi	1986	4,700	43	−	R	−	C	III	−	+	10	56
Huerga (33)	Liberia	2005	1,034	N/A	+	R	−	C	III	−	+	20	69
Ike (48)	Nigeria	1998–2003	7,399	42	−	R	−	C	III	−	+	18	56
Kakembo (34)	S. Africa	1994	2,142	45	+	R	+	M	?	−	−	18	50
Lester (25)	Ethiopia	1971–75	4,640	33	+	P	+	C	III	+	+	36	100
Marszalek (38)	S. Africa	2001–2003	462	48	−	R	+	C	III	−	−	18	50
Mudiayi (39)	Zimbabwe	1987–94	12,280	46	−	R	+	C	III	−	−	18	50
Ndjeka (45)	S. Africa	1996	1,486	55	−	R	−	?	?	−	−	10	28
Odenigbo (81)	Nigeria	2005–2007	1,860	46	−	R	−	C	III	−	+	20	56
Osuafor (82)	Nigeria	1990–92	530	47	−	R	−	C	III	−	−	15	42
Patel (30)	Uganda	1966–68	6,154	36	−	P	−	C	III	−	+	21	58
Pavlica (41)	Ethiopia	1966–70	3,922	20	−	R	−	C	III	+	−	20	56
Pobee (49)	Ghana	1971–72	462	30	−	R	−	C	III	−	+	20	56
Reeve (46)	S. Africa	1982–83	997	48	−	R	−	?	?	−	−	10	28
Shaper (43)	Uganda	1957	2,466	33	−	R	−	C	III	+	+	25	69
Tambwe (83)	DRC	1988–89	1,624	N/A	−	R	−	C	II	−	+	17	47
Turner (31)	Kenya	1960	2,006	26	−	R	−	C	III	−	+	20	56
Walker (40)	S. Africa	1991	2,567	60	−	R	+	M	III	−	−	16	44
Williams (32)	Uganda	1951–78	10,498	44	−	R	+	C	II	+	+	25	69

a Individual studies were assessed for the presence of each criterion and if present, the maximum score for that criterion awarded. If the criterion was absent, one point was awarded. An intermediate score (50% of maximum) was assigned when the characteristic of interest was unclear (?), for category M under cadre of staff and category II under diagnostic facilities. +/− Characteristic was present/absent in the study. ? – Presence/absence of characteristic was unclear. R/P – study was retrospective (R)/prospective (P) in design. C/M – Consultants/Medical Officers. N/A – Not available/reported.

### Methodological quality

The median score for all the studies after applying weighted points to each aspect of the studies (Table 1) was 55% (IQR 47.2–63.9). Only one study (25) satisfied all criteria on methodological quality.

#### Study design

Only 8 (27%) studies were prospective in nature, the most recent one having been completed in 1995 (25–32). Five of these prospective studies were conducted in university teaching hospitals that had high-level staff and relatively advanced diagnostic facilities. However, given their urban location the results of these studies may not have been representative of the disease pattern in the majority of the population in the countries studied, a fact acknowledged by all the authors.

#### Use of standard case definitions

Four studies (13%) provided case definitions for the illnesses that were studied (25, 26, 33, 34). One study conducted in Uganda (30) provided a case definition for only one condition (anemia) but this differed from the standard WHO definition (35).

#### Use of international classification of diseases coding

All of the studies categorized illnesses into various bodily systems as a result of the multiplicity of diagnoses. However, only one third of the studies (25, 32, 34, 36–41) used the WHO recommended ICD system that enables comparisons between studies and aggregation of data across different sites. In one study the coding of illnesses into ICD categories was done retrospectively (32).

#### Admission criteria

Thirteen studies mentioned the presence of admission criteria for patients to the hospitals where the studies were conducted but only five specified what these criteria were. Two of these studies were conducted in the same hospital at different time points, thus reducing the number of hospitals in which admission criteria were reported to four. In the five reports where admission criteria were described (25, 30, 32, 42, 43), they consisted of seriousness of the illness, referral from lower level facilities, and suitability for teaching and research with patients not meeting any of these criteria being referred to other facilities. The remaining reports did not specify what the admission criteria were.

#### Cadre of hospital staff

Most of the studies were conducted in hospitals that had consultants as the highest level of staff, and these were responsible for determining the final diagnosis given to patients. In four studies, all of which were conducted in rural areas, Medical Officers served as the highest cadre of medical staff (29, 34, 40, 44). In two studies, the level of training of the staff at the hospitals involved was not reported (45, 46).

#### Reporting on diagnostic facilities

We postulated that hospitals located in urban areas such as university teaching hospitals would have more advanced diagnostic facilities. Of the 14 studies carried out in university/teaching hospitals nine reported having class III diagnostic facilities (26–28, 30, 42, 43, 47–49). These included more advanced imaging, pathology, and biochemical tests. In three studies, autopsies were performed on more than 50% of deaths (30, 43, 49). Only one study carried out in a rural hospital had advanced diagnostic facilities thus limiting the range and accuracy of diagnoses for rural studies (40).

#### Reporting on case fatality rates

We found 18 (60%) studies that either reported case fatality rates for individual illnesses or overall in-hospital mortality for patients observed during the study periods. Three quarters of the studies giving mortality rates were conducted in hospitals located in urban areas.

### Leading causes of hospital admission

**Fig. 3:**
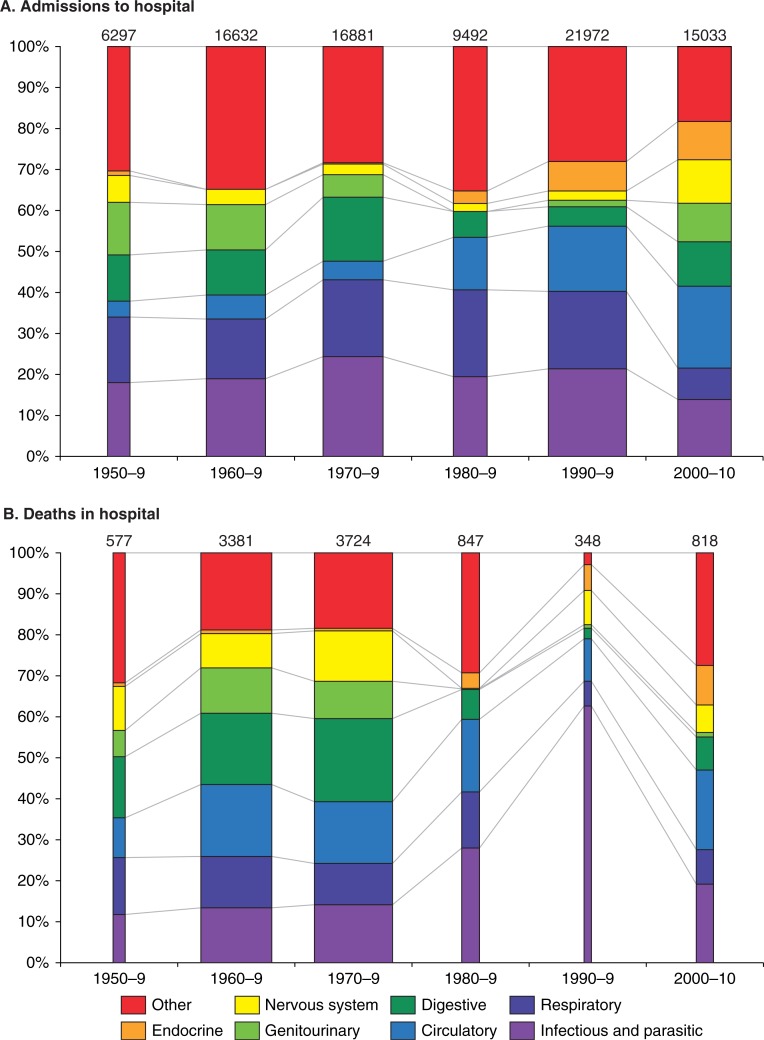
Temporal distribution of admissions and deaths by ICD diagnostic category* A) Frequency of admissions per decade by ICD diagnostic category. B) Frequency of deaths per decade by ICD diagnostic category.

Note: For each decade the leading diagnostic categories for both admissions and deaths are shown. The width of the columns is proportional to the amount of observations available for each decade. Detailed data regarding all causes of admission and death for each period can be found in the supplementary appendix.

We found 27 studies reporting data on 86,307 medical admissions for the period from 1950–2010 (Table S2). The proportions of admissions and deaths by ICD diagnostic category over the period are shown in Fig. 3. Infectious and parasitic diseases, including malaria, bacterial diseases, and HIV disease, were the leading cause of admission over the period accounting for 19.8% (95% CI 19.6–20.1) of all admissions. Respiratory illnesses were second accounting for 16.2% (95% CI 16.0–16.5) of admissions while diseases of the circulatory system were third at 11.3% (95% CI 11.0–11.5). When we restricted the analysis to admissions from studies that ranked above the 75th percentile in the methodological score, infectious and parasitic diseases, and respiratory illnesses remained the leading causes of admission, accounting for 27.4% (95% CI 26.9–27.9) and 18.4%(95% CI 18.0–18.9) of all admissions. The remaining leading causes of admission were in descending order: digestive system 13.9% (95% CI 13.6–14.3), genitourinary system 10.6% (95% CI 10.3–11.0) and circulatory system disorders 7.4% (95% CI 7.1–7.7). The class of disease among all admissions varied with time in decades (χ^2^=14,000, df 30; p<0.0005). The proportion of admissions due to circulatory system disorders increased five-fold over the period from 3.9% of admissions in 1950–59 to 19.9% of admissions in 2000–2010 (RR 5.1, χ^2^ test for trend p<0.00005). Infectious and parasitic diseases decreased from 18.2% to 13.9% of all admissions over the period, a 24% decrease (RR 0.76, 95% CI 0.71–0.82). Respiratory illnesses decreased from 16.2% of all admissions to 7.7% (RR 0.47, 95% CI 0.44–0.51). Endocrine and nervous system disorders increased significantly over the period, although each accounted for less than 5% of all admissions (Table S2).

*Some studies compared diseases over two time periods, both periods were included. Observations for studies that were conducted over several decades were equally split over the duration of the study if the temporal distribution of admissions/deaths had not been reported.

### Leading causes of in-hospital mortality

We obtained in-hospital cause of death data for 9,695 patients from 15 studies for the period 1950–2010 (Table S2, Fig. 3). Infectious and parasitic diseases, disorders of the circulatory system, and digestive system disorders were the leading causes of death accounting for 17.1% (95% CI 16.4–17.9), 16.0% (95% CI 15.3–16.8), and 16.2% (95% CI 15.4–16.9) of all deaths, respectively. The class of disease among all deaths varied with time in decades (χ^2^=1100, df 30; p<0.0005). The proportion of deaths caused by disorders of the circulatory system rose from 9.7% in 1950–59 to 19.4% in 2000–2010 (RR 2.0, 95% CI 1.5–2.7, χ^2^ test for trend p=0.08). Deaths caused by infectious and parasitic diseases rose from 11.8% in 1950–9 to 19.2% in 2000–2010 (RR 1.6, 95% CI 1.3–2.1). Comparison of the proportion of deaths caused by digestive system disorders at the beginning of the study period and at the end showed a decrease from 14.9% to 8.1% (RR 0.54, 95% CI 0.40–0.73). Deaths due to disorders of the endocrine, nervous, and genitourinary systems all showed significant decreases (Table S2), but the combined contribution of these diagnostic categories to overall mortality was less than 20%. When we restricted the analysis of causes of death to studies that ranked above the 75th percentile on the methodological score, we observed no meaningful changes in the result.

Case fatality rates for the entire period for different disease categories could not be calculated because of inconsistent reporting on the number of patients admitted with each medical condition. Two recent retrospective studies reported case fatality rates and the diagnostic categories with the highest case fatality rates were infectious and parasitic diseases (25%) and circulatory illnesses (21%) (25, 33).

## Discussion

We found 30 eligible studies conducted in 10 sub-Saharan countries and covering a 61-year time period. In most African countries in there are no published data on hospital deaths (Fig. 2) and our illustration of research activity trends (Fig. 3) provides no evidence that this situation is improving over time. All of this is in spite of the fact that Africa bears nearly a quarter of the world's burden of disease (50).

Despite the limitations identified in the few studies obtained, as indicated by the low methodology scores, some trends were discernible; for example, the proportion of admissions due to cardiovascular illnesses, composed mainly of stroke and heart failure, were found to have increased significantly over the study period. The proportion of admissions due to infectious and parasitic diseases decreased over the study period. However, the proportion of deaths caused by infectious and parasitic diseases increased during the period, which roughly coincides with the emergence of the HIV epidemic. HIV-related illnesses contributed the most to the burden of infectious disease over the past 10 years with a very high case fatality, suggesting that late presentation, due to limited access to HIV testing facilities, continues to be a significant problem on the continent (51). HIV may also increase the risk of heart diseases such as cardiomyopathy (52) and cancers (53) providing one illustration of the links between communicable and non-communicable diseases. Due to the limitations of the data, it is difficult to determine whether or not the observed trends were a result of the ongoing epidemiological transition with an increased burden of non-communicable illnesses in the continued presence of HIV and other infectious diseases. The observed changes could be due to changing hospital admission policies, improved hospital access for certain age or disease groups or new treatment availability, factors that we were unable to examine. These would be acting in concert with other explanations for the increasing burden of non-communicable disease in sSA, including demographic changes resulting in an increase in the number of children surviving to adulthood (4) and an increase in the prevalence of cardiovascular risk factors such as hypertension and diabetes (54, 55). Consistent with these observations as well as our results, community-based data from South Africa showed an increase in non-communicable diseases as a cause of death between 1992 and 2005 (56). This was also accompanied by a large increase in HIV-related deaths (56). However, when compared to estimates based on the global burden of disease model (2), which is itself imperfect (57), our results suggest a bigger contribution from non-communicable diseases. These contrasting results emphasize the importance of defining patterns of disease accurately as the transition proceeds in order to allocate scarce health resources. Research activities and funding also need to be guided by the burden of disease but evidence from the developing world suggests that this does not always happen (58, 59).

This review also exposes the methodological deficiencies of the few studies that have contributed data, an issue that has received little attention (11). The most common shortcoming was the failure to use standard case definitions for the illnesses described. This is attributable to the fact that most of the studies were retrospective in design and it would have been difficult to access records for purposes of verifying diagnoses listed in the discharge records. Without a clear, measurable, and specific definition of an illness, consumers of reports are unable to interpret, compare, and aggregate data from various studies (22). An example of the distortion that can arise from the use of different case definitions was the ‘surge’ in AIDS cases that arose after the Centers for Disease Control and Prevention (CDC) expanded their case definition for AIDS in 1993 (60). The WHO has established case definitions for the most common illnesses such as anemia (35), pneumonia (61), stroke (62), and diabetes mellitus (63). While it might be argued that some of the case definitions did not exist at the time when the earlier studies were conducted, only one of the more recent studies reviewed reported the use of case definitions (33). Obviously, improving the diagnostic capabilities of facilities would permit the use of more sophisticated and differentiating case definitions that rely upon test results.

The failure of two-thirds of the studies reviewed to use standard ICD coding systems also limits our capacity to compare studies or aggregate data. Stroke, for example, was classified as a neurological disorder in some studies while other studies classified it as a cardiovascular disorder in concordance with the ICD coding system. Diarrhea was listed as a digestive system illness in some studies while in others it was classified as an infectious and parasitic disease.

In many countries in sSA diagnostic facilities can only be found in large referral hospitals located in urban areas (64). Our analysis found that most of the studies describing medical illnesses in adults had been conducted in university teaching hospitals and referral facilities located in urban areas. These were also the facilities most likely to be operated by consultants (65) who, by virtue of their more advanced training, are more likely to publish research findings, potentially biasing the sample of reports available for this review. At the time that they were published, most of the population of sSA was living in rural areas with limited access to tertiary referral hospitals thus limiting the generalizability of the findings (20). As the pace of urbanization on the continent increases (66), studies conducted in urban hospitals are likely to be more representative than in the past.

The demographic transition that is driving this urbanization, with its associated increase in endemic non-communicable diseases, argues for substantial improvements in the diagnostic and curative services in adult health in sSA to enable the correct classification of disease and early initiation of control measures, especially for cardiovascular and other non-communicable chronic illnesses (67). Initiatives such as the WHO-supported Health Metrics Network (68) that aim to improve the collection of health-related data in developing countries by providing guidelines and templates need to appreciate that the quality of clinical data from hospitals in sSA will compromise the utility of these systems unless there is substantial investment in instruments and training. Adult demographic surveillance networks, such as the INDEPTH (69) network, could also provide adult morbidity data by linking the data from dedicated hospital surveillance teams to their population registers. Ultimately, well-conducted cohort studies will be needed, where the outcomes (and exposure variables) are compliant with international standards of disease detection and classification, in order to identify risk factors for important illnesses in sSA (70).

Our review had two principal limitations. Many articles publishing findings on research in Africa are not available through the Internet (71). Organizations such as AJOL (www.ajol.info) and HINARI (http://www.who.int/hinari/en/) have succeeded in providing substantial access to publications from Africa online. However, many publications are not available online and we were obliged to access print copies of 20 papers from the British Library and could not find a suitable source for a further six papers. Despite good electronic database we found 20% of the relevant papers through hand rather than online searching. Our exclusion of papers written in languages other than English, which were even more difficult to obtain, also potentially biased our results in favor of English-speaking countries. Despite these shortcomings we believe that the studies reviewed here are a fair representation of the research articles that have been published on the subject in the past six decades.

For some disease categories, such as neoplasms, we found extremely sparse data, which we attribute to the lack of appropriate diagnostic facilities rather than to insignificant numbers of patients with these diseases. We also observed an uneven contribution from different regions of the continent to the data through the different time periods studied, most likely due to variations in the availability of health-reporting infrastructure as a result of political or economic instability.

## Conclusions and recommendations for the conduct of future studies

We found that there were very limited data on the causes of hospital admissions and death among adults in sSA, and these data had significant limitations. However, the review provides evidence to suggest that cardiovascular diseases account for a significant and increasing fraction of the causes of hospitalization among adults in sSA. As the epidemiology of adult disease in sSA transitions from infectious to non-communicable diseases, health researchers and policy makers will need to establish reliable and consistent systems for diagnosing and recording disease in adults in order to optimize treatments and preventive interventions.

## Supplementary appendix

**Table S1:** Studies excluded from review

Reason for exclusion	Study
Unable to extract data on adults	Accorsi S, Kedir N, Farese P, Dhaba S, Racalbuto V, Seifu A, et al. Poverty, inequality and health: the challenge of the double burden of disease in a non-profit hospital in rural Ethiopia. Transactions of the Royal Society of Tropical Medicine and Hygiene. 2009 May; 103: 461–8.
	Petit P. Analysis of hospital records in four African countries, 1975–1990, with emphasis on infectious diseases. Journal of Tropical Medicine and Hygiene. 1995; 98217–27.
	Feikin DR, Adazu K, Obor D, Ogwang S, Vulule J, Hamel MJ, et al. Mortality and health among internally displaced persons in western Kenya following post-election violence, 2008: novel use of demographic surveillance. Bulletin of the World Health Organization. 2010; 88: 601–8.
Conducted during period of war	Accorsi S, Fabiani M, Nattabi B, Ferrarese N, Corrado B, Iriso R, et al. Differences in hospital admissions for males and females in northern Uganda in the period 1992–2004: a consideration of gender and sex differences in health care use. Transactions of the Royal Society of Tropical Medicine and Hygiene 2007; 101: 929–38.
	Accorsi S, Fabiani M, Nattabi B, Corrado B, Iriso R, Ayella EO, et al. The disease profile of poverty: morbidity and mortality in northern Uganda in the context of war, population displacement and HIV/AIDS. Transactions of the Royal Society of Tropical Medicine and Hygiene. 2005; 99: 226–33.
	Accorsi S. The increasing burden of infectious diseases on hospital services at St Mary's Hospital Lacor, Gulu, Uganda. The American Journal Of Tropical Medicine And Hygiene 2001; 64154–8.
Focused on a single cause of admissions	Abe E, Omo-Aghoja LO. Maternal mortality at the Central Hospital, Benin City Nigeria: a ten year review. African Journal Of Reproductive Health. 2008; 12: 17–26.
	Arthur G, Bhatt SM, Muhindi D, Achiya GA, Kariuki SM, Gilks CF. The changing impact of HIV/AIDS on Kenyatta National Hospital, Nairobi from 1988/89 through 1992 to 1997. AIDS 2000 ; 14: 1625–31.
	Ferrand RA, Bandason T, Musvaire P, Larke N, Nathoo K, Mujuru H, et al. Causes of acute hospitalization in adolescence: burden and spectrum of HIV-related morbidity in a country with an early-onset and severe HIV epidemic: a prospective survey. PLoS Medicine 2010; 7: e1000178.
	Floyd K. Admission Trends in a rural South African hospital during the early years of the HIV epidemic. JAMA 1999; 282: 1087–91.
	Harries A, Mvula B. The changing pattern of mortality in an African medical ward. Tropical and Geographical Medicine 1995; 47171–4.
	Reid A, Dedicoat M, Lalloo D, Gilks CF. Trends in adult medical admissions in a rural South African hospital between 1991 and 2002. Journal of Acquired Immune Deficiency Syndromes (1999) 2005 ; 40: 53–6.
	Teklu B. Pattern of respiratory diseases in a general hospital in Addis Ababa. Ethiop Med J 1980; 18135–43.
	Onyekonwu CG, Umeh RE. Pattern of Ocular Diseases among Computer users in Enugu, Nigeria. Orient Journal of Medicine 2007.
	Okoye BCC, Onotai LO. Pattern of geriatric otolaryngological diseases in Port Harcourt. Nigerian Journal of Medicine 2007.
	Onyenekwe C, Meludu S, Dioka C. Pattern and distribution of sexually transmitted diseases in Lagos Nigeria. Journal of Biomedical Investigation 2004.
	Colvin M, Dawood S, Kleinschmidt I, Mullick S, Lallo U. Prevalence of HIV and HIV-related diseases in the adult medical wards of a tertiary hospital in Durban, South Africa. Int J STD AIDS 2001; 12: 386–9.
	De Cock KM, Barrere B, Lafontaine MF, Diaby L, Gnaore E, Pantobe D, et al. Mortality trends in Abidjan, Côte d'Ivoire, 1983–1988. AIDS 1991; 5: 393.
	Masiira-Mukasa N, Ombito BR. Surgical admissions to the Rift Valley Provincial General Hospital, Kenya. East Afr Med J 2002; 79: 373–8.
Focused on a single age sub-group	Mets TF. The disease pattern of elderly medical patients in Rwanda, central Africa. The Journal of tropical medicine and hygiene. 1993; 96: 291–300.
	Ferrand RA, Bandason T, Musvaire P, Larke N, Nathoo K, Mujuru H, et al. Causes of acute hospitalization in adolescence: burden and spectrum of HIV-related morbidity in a country with an early-onset and severe HIV epidemic: a prospective survey. PLoS Medicine 2010; 7: e1000178.
	Olubuyide I, Hart P, Alli-Gombe A, Adesanya J, Okosieme T, Otunla T, et al. Diseases of the middle-aged population: a study of 4,184 patients. Central African Journal of Medicine 1991.
	Sanya E, Akande T, Opadijo G, Olarinoye J, Bojuwoye B. Pattern and outcome of medical admission of elderly patients seen at University of Ilorin Teaching Hospital, Ilorin. African Journal Of Medicine And Medical Sciences 2008; 37375–81.
	Mohamed EY, Abdelfadil L, Ziyada MM, Abdelgadir MA, Khalid A, Ashmaig AL. The pattern of obstetrical and gynaecological admissions in Ribat University Hospital, Khartoum. Sudanese Journal of Public Health 2006; 1: 112–116.
	Okot-Nwang, M, Wabwire-Mangen F, Kagezi VB. Increasing prevalence of tuberculosis among Mulago Hospital admissions, Kampala, Uganda (1985–1989). Tuber Lung Dis 1993; 74: 121–5.
	McLigeyo SO. The pattern of geriatric admissions in the medical wards at the Kenyatta National Hospital. East Afr Med J 1993; 70: 37–9.

**Table S2:** Numbers of admissions and deaths for each decade*

Number of deaths by disease category 1950–2010^a^						

	Period					
	
Disease Category	1950–59	1960–69	1970–79	1980–89	1990–99	2000–10
Infectious and parasitic diseases	68 (12%)	453 (13%)	527 (14%)	237 (28%)	218 (63%)	157 (19%)
Respiratory	80 (14%)	424 (13%)	375 (11%)	116 (14%)	21 (6%)	69 (8%)
Circulatory	56 (10%)	593 (18%)	561 (17%)	150 (18%)	36 (10%)	159 (19%)
Nervous system	62 (11%)	283 (8%)	458 (14%)	1 (0%)	29 (8%)	55 (7%)
Digestive	86 (15%)	588 (17%)	755 (22%)	63 (7%)	9 (3%)	66 (8%)
Endocrine	5 (1%)	30 (1%)	23 (1%)	32 (4%)	22 (6%)	79 (10%)
Genitourinary	37 (6%)	374 (11%)	339 (10%)	0 (0%)	3 (1%)	9 (1%)
Other	183 (3%)	636 (19%)	686 (20%)	248 (29%)	10 (3%)	225 (28%)
Total deaths	577 (100%)	3,381 (100%)	3,724 (100%)	847 (100%)	348 (100%)	818 (100%)

Number of admissions by disease category 1950–2010^b^						

	Period					
	
Disease Category	1950–59	1960–69	1970–79	1980–89	1990–99	2000–10

Infectious and parasitic diseases	1,147 (18%)	3,153 (19%)	4,125 (24%)	1,905 (20%)	4,696 (21%)	2,090 (14%)
Respiratory	1,018 (16%)	2,422 (15%)	3,172 (19%)	2,074 (22%)	4,151 (19%)	1,151 (8%)
Circulatory	247 (4%)	974 (6%)	768 (5%)	1,253 (13%)	3,495 (16%)	3,001 (20%)
Nervous system	417 (7%)	619 (4%)	438 (3%)	192 (2%)	499 (2%)	1,595 (11%)
Genitourinary	817 (13%)	1,839 (11%)	932 (6%)	0 (0%)	347 (2%)	1,412 (9%)
Digestive	718 (11%)	1,829 (11%)	2,644 (16%)	618 (7%)	1,042 (5%)	1,631 (11%)
Endocrine	70 (1%)	0 (0%)	54 (0%)	298 (3%)	1,578 (7%)	1,402 (9%)
Other	1,933 (31%)	5,795 (35%)	4,802 (28%)	3,450 (36%)	6,165 (28%)	2,751 (18%)
Total admissions	6,297 (100%)	16,632 (100%)	16,881 (100%)	9,492 (100%)	21,972 (100%)	15,033 (100%)

* Column totals may not add up to exactly 100% due to rounding.

a Data for deaths was extracted from references: (25–28, 30, 32, 33, 36, 37, 43, 49, 78, 80, 82).

b Data on admissions was extracted from references: (25–31, 33, 34, 37–46, 48, 77–83).

## References

[1] Phillips M, Feachem RG, Murray CJ, Over M, Kjellstrom T (1993). Adult health: a legitimate concern for developing countries. Am J Public Health.

[2] Jamison DT, Feachem RG, Malegapuru WM, Bos ER, Baingana FK (2006). Disease and mortality in sub-Saharan Africa.

[3] World Bank World development report 1993 – investing in health. http://files.dcp2.org/pdf/WorldDevelopmentReport1993.pdf.

[4] Rajaratnam JK, Marcus JR, Flaxman AD, Wang H, Levin-rector A, Dwyer L (2010). Neonatal, postnatal, childhood, and under-5 mortality for 187 countries, 1970–2010: a systematic analysis of progress towards millennium development goal 4. Lancet.

[5] You D, Jones G, Hill K, Wardlaw T, Chopra M (2010). Levels and trends in child mortality, 1990–2009. Lancet.

[6] United Nations Department of Economic and Social Affairs (2009). World population prospects: the 2008 revision. http://www.un.org/esa/population/publications/popnews/Newsltr_87.pdf.

[7] UNAIDS, WHO, UNICEF (2004) Children on the brink 2004: a joint report on orphan estimates and program strategies. http://data.unaids.org/publications/.../unicef_childrenonthebrink2004_en.pdf.

[8] Kitange HM, Machibya H, Black J, Mtasiwa DM, Masuki G, Whiting D (1996). Outlook for survivors of childhood in sub-Saharan Africa: adult mortality in Tanzania. Adult morbidity and mortality project. BMJ.

[9] Mathers CD, Fat DM, Inoue M, Rao C, Lopez AD (2005). Counting the dead and what they died from: an assessment of the global status of cause of death data. Bull World Health Organ.

[10] Rajaratnam JK, Marcus JR, Levin-rector A, Chalupka AN, Wang H, Dwyer L (2010). Worldwide mortality in men and women aged 15–59 years from 1970 to 2010: a systematic analysis. Lancet.

[11] Lopez AD, Mathers CD, Ezzati M, Jamison DT, Murray CJL (2006). Global and regional burden of disease and risk factors, 2001: systematic analysis of population health data. Lancet.

[12] Koyanagi A, Shibuya K (2010). What do we really know about adult mortality worldwide?. Lancet.

[13] Cooper RS, Osotimehin B, Kaufman JS, Forrester T (1998). Disease burden in sub-Saharan Africa: what should we conclude in the absence of data?. Lancet.

[14] WHO (2010). Global status report on noncommunicable diseases. http://www.who.int/nmh/publications/ncd_report2010/en/.

[15] Maher D, Smeeth L, Sekajugo J (2010). Health transition in Africa: practical policy proposals for primary care. Bull World Health Organ.

[16] Beaglehole R, Bonita R, Horton R, Adams C, Alleyne G, Asaria P (2011). Priority actions for the non-communicable disease crisis. Lancet.

[17] Law T, Lavis J, Hamandi A, Cheung A, El-Jardali F (2012). Climate for evidence-informed health systems: a profile of systematic review production in 41 low-and middle-income countries, 1996–2008. J Health Res Policy.

[18] Beaglehole R, Horton R (2010). Chronic diseases: global action must match global evidence. Lancet.

[19] Moisi JC, Nokes DJ, Gatakaa H, Williams TN, Bauni E, Levine OS (2011). Sensitivity of hospital-based surveillance for severe disease: a geographic information system analysis of access to care in Kilifi district, Kenya. Bull World Health Organ.

[20] Arcury TA, Gesler WM, Preisser JS, Sherman J, Spencer J, Perin J (2005). The effects of geography and spatial behavior on health care utilization among the residents of a rural region. Health Serv Res.

[21] WHO (2004). International statistical classification of diseases and health related problems. http://apps.who.int/classifications/apps/icd/icd10online/.

[22] Grimes DA, Schulz KF (2002). Descriptive studies: what they can and cannot do. Lancet.

[23] Hennekens C, Buring J (1987). Epidemiology in medicine.

[24] Vandenbroucke JP, von Elm E, Altman DG, Gotzsche PC, Mulrow CD, Stuart J (2007). Strengthening the reporting of observational studies in epidemiology (STROBE): explanation and elaboration. PLoS Medicine.

[25] Lester FT (1976). The Pattern of adult medical admissions in Addis Ababa, Ethiopia. East Afr Med J.

[26] Amsel M, Matewos A (1999). The changing pattern of diseases in the mid 1990's: experience of a teaching hospital in North Western Ethiopia. Ethiop J Health Dev.

[27] Barr RD (1972). A two-year prospective analysis of emergency admissions to an adult medical unit at the Kenyatta National Hospital, Nairobi. East Afr Med J.

[28] Brown KGE (1975). Analysis of admissions to the adult medical wards at Queen Elizabeth Central Hospital, Blantyre, Malawi. East Afr Med J.

[29] Edginton ME, Hodkinson J, Seftel HC (1972). Disease patterns in a South African rural Bantu population, including a commentary on comparisons with the pattern in urbanized Johannesburg Bantu. S Afr Med J.

[30] Patel KM, Lwanga SK (1971). A study of medical admissions to Mulago Hospital, Kampala. East Afr Med J.

[31] Turner PP (1962). The pattern of disease as seen by medical admissions to the Coast Province General Hospital in 1960. East Afr Med J.

[32] Williams E, Hayes R, Smith P (1986). Admissions to a rural hospital in the West Nile District of Uganda over a 27-year period. J Trop Med Hyg.

[33] Huerga H, Vasset B, Prados E (2009). Adult and paediatric mortality patterns in a referral hospital in Liberia 1 year after the end of the war. Trans R Soc Trop Med Hyg.

[34] Kakembo A, Walker B, Walker A (1996). Causes of admission of African patients to Gelukspan Hospital. East Afr Med J.

[35] McLean E, Cogswell M, Egli I, Wojdyla D, de Benoist B (2009). Worldwide prevalence of anaemia. WHO vitamin and mineral nutrition information system, 1993–2005. Public Health Nutr.

[36] Adekunle O, Olatunde I, Abdullateef R (2008). Causes and pattern of death in a tertiary health institution in south western Nigeria. Niger Postgrad Med J.

[37] Dean MP, Gear JS (1986). Medical admissions to Hillbrow Hospital, Johannesburg, by discharge diagnosis. S Afr Med J.

[38] Marszalek J, De Villiers PJT (2006). Morbidity profile of admissions to GF Jooste Hospital, Manenberg, Cape Town. S A Fam Pract.

[39] Mudiayi TK, Onyanga-Omara A, Gelman ML (1997). Trends of morbidity in general medicine at United Bulawayo Hospitals, Bulawayo, Zimbabwe. Cent Afri J Med.

[40] Walker ARP, Walker BF, Dunn MJ, Dunn SE (1994). Causes of admissions of rural African patients to Murchison Hospital, Natal, South Africa. J R Soc Health.

[41] Pavlica D (1970). Analysis of medical admissions to the Armed Forces Hospital in Addis Ababa from January 1966 to January 1970. Ethiop Med J.

[42] Adetuyibi A, Akisanya JB, Onadeko BO (1976). Analysis of the causes of death on the medical wards of the University College Hospital, Ibadan over a 14-year period (1960–1973). Trans R Soc Trop Med Hyg.

[43] Shaper AG, Shaper L (1958). Analysis of medical admissions to Mulago Hospital, 1957. East Afr Med J.

[44] Griffiths ML (1981). A comparison of admissions to a semirural hospital between the years 1959/1960 and 1977/1978. S Afr Med J.

[45] Ndjeka N, Ogunbanjo G (2004). Disease patterns in the medical wards of a rural South African hospital. SA Fam Pract.

[46] Reeve PA, Falkner MJ (1986). Disease patterns in a rural black population. S Afr Med J.

[47] Akoria O, Unuigbe E (2010). A 6-month review of medical admissions in a Nigerian teaching hospital. Int J Health Res.

[48] Ike S (2008). The pattern of admissions into the medical wards of the University of Nigeria Teaching Hospital, Enugu (2). Niger J Clin Pract.

[49] Pobee JO (1976). A review of the causes of death in adult medical wards of Korle Bu Teaching Hospital, Accra, Ghana. Afr J Med Sci.

[50] WHO (2006). Working together for health; the world health report 2006. http://www.who.int/whr/2006/en/.

[51] Kigozi IM, Dobkin LM, Martin JN, Geng EH, Muyindike W, Emenyonu NI (2009). Late-disease stage at presentation to an HIV clinic in the era of free antiretroviral therapy in Sub-Saharan Africa. J Acquir Immune Defic Syndr.

[52] Barbarinia G, Barbaro G (2003). Incidence of the involvement of the cardiovascular system in HIV infection. AIDS.

[53] Kesselring A, Gras L, Smit C, van Twillert G, Verbon A, de Wolf F (2011). Immunodeficiency as a risk factor for non-AIDS-defining malignancies in HIV-1-infected patients receiving combination antiretroviral therapy. Clin Infect Dis.

[54] Kearney PM, Whelton M, Reynolds K, Muntner P, Whelton PK, He J (2005). Global burden of hypertension: analysis of worldwide data. Lancet.

[55] Mbanya JC, Motala AA, Sobongwi E, Assah FK, Enoru ST (2010). Diabetes in sub-Saharan Africa. Lancet.

[56] Tollman SM, Kahn K, Sartorius B, Collinson MA, Clark SJ, Garenne ML (2008). Implications of mortality transition for primary health care in rural South Africa: a population-based surveillance study. Lancet.

[57] Byass P (2010). The imperfect world of global health estimates. PLoS Medicine.

[58] Perel P, Miranda JJ, Ortiz Z, Casas JP (2008). Relation between the global burden of disease and randomized clinical trials conducted in Latin America published in the five leading medical journals. PLoS ONE.

[59] Sridhar D, Batniji R (2008). Misfinancing global health: a case for transparency in disbursements and decision making. Lancet.

[60] Anonymous (1993). Impact of the expanded AIDS surveillance case definition on AIDS case reporting – United States, first quarter, 1993. MMWR Morb Mortal Wkly.

[61] International Working Group (2002). Integrated management of adolescent and adult illness (IMAI). http://www.who.int/hiv/pub/imai/en/index.html.

[62] Hatano S (1976). Experience from a multicenter stroke register: a preliminary report. Bull World Health Organ.

[63] World Health Organization (2006) Definition and diagnosis of diabetes mellitus and intermediate hyperglycemia: a report of a WHO/IDF consultation. http://whqlibdoc.who.int/publications/2006/9241594934_eng.pdf.

[64] Adeyi OA (2011). Pathology Services in developing countries: the west African experience. Arch Pathol Lab Med.

[65] Lemiere C, Herbst CH, Jahanshahi N, Smith E (2011). Reducing geographical imbalances of health workers in Sub-Saharan Africa: a labor market perspective on what works, what does not, and why. http://www-wds.worldbank.org/external/default/WDSContentServer/WDSP/IB/2011/01/07/000333038_20110107020314/Rendered/PDF/588430NWP0Heal101public10BOX353816B.pdf.

[66] UN Habitat (2004). State of the world's cities: trends in sub-Saharan Africa. http://ww2.unhabitat.org/mediacentre/sowckit.asp.

[67] Damasceno A, Cotter G, Dzudie A, Sliwa K, Mayosi BM (2007). Heart failure in sub-Saharan Africa: time for action. J Am Coll Cardiol.

[68] WHO/Health Metrics Network (2008). Framework and standards for country health information systems. http://www.who.int/healthmetrics/documents/hmn_framework200803.pdf.

[69] Kowal P, Kahn K, Ng N, Naidoo N, Abdullah S, Bawah A (2011). Ageing and adult health status in eight lower-income countries: the INDEPTH WHO-SAGE collaboration. Glob Health Action.

[70] Holmes MD, Dalal S, Volmink J, Adebamowo CA, Njelekela M, Fawzi F (2010). Non-communicable diseases in Sub-Saharan Africa: the case for cohort studies. PLoS Medicine.

[71] Pakenham-Walsh N, Priestley C (2002). Towards equity in global health knowledge. QJM.

[72] Reyburn H, Mbatia R, Drakeley C, Carneiro I, Mwakasungula E, Mwerinde O (2004). Overdiagnosis of malaria in patients with severe febrile illness in Tanzania: a prospective study. BMJ.

[73] WHO (1953). Manual of the international statistical classification of diseases, injuries, and causes of death. Bull World Health Org.

[74] Mullan F, Frehywot S, Omaswa F, Buch E, Chen C, Greysen SR (2010). Medical schools in sub-Saharan Africa. Lancet.

[75] Rich EC, Gifford G, Luxenberg M, Dowd B (1990). The relationship of house staff experience to the cost and quality of inpatient care. JAMA.

[76] Mullan F, Frehywot S (2007). Non-physician clinicians in 47 sub-Saharan African countries. Lancet.

[77] Agomuoh DI, Unachukwu CN (2008). Pattern of diseases among medical admissions in Port Harcourt, Nigeria. Niger Med Pract.

[78] Bardgett HP, Beeching NJ (2006). Increase in hospital mortality from non-communicable disease and HIV-related conditions in Bulawayo, Zimbabwe, between 1992 and 2000. Trop Doct.

[79] Gill GV (1995). Changing patterns of medical disease in Soweto, South Africa 1981–1990. Trop Doct.

[80] Harries AD, Richard S, Wirima J (1990). Medical admissions to Kamuzu Central Hospital, Lilongwe Malawi in 1986: comparison with admissions to Queen Elizabeth Central Hospital, Blantyre in 1973. Trop Geogr Med.

[81] Odenigbo C, Oguejiofor O (2009). Pattern of medical admissions at the Federal Medical Centre, Asaba – a two year review. Niger J Clin Pract.

[82] Osuafor TO, Ele PU (2004). The pattern of admissions in the medical wards of Nnamdi Azikiwe University Teaching Hospital (NAUTH) Nnewi. Orient J Med.

[83] Tambwe M, Mbala M, Lusamba DN, M'buyamba-Kabangu JR (1995). Morbidity and mortality in hospitalised Zairean adults. S Afr Med J.

